# Vegetable Grafting as a Tool to Improve Drought Resistance and Water Use Efficiency

**DOI:** 10.3389/fpls.2017.01130

**Published:** 2017-06-30

**Authors:** Pradeep Kumar, Youssef Rouphael, Mariateresa Cardarelli, Giuseppe Colla

**Affiliations:** ^1^Central Arid Zone Research Institute (ICAR),Jodhpur, India; ^2^Department of Agricultural Sciences, University of Naples Federico IINaples, Italy; ^3^Consiglio per la Ricerca in Agricoltura e l’Analisi dell’Economia Agraria, Centro di Ricerca Agricoltura e AmbienteRome, Italy; ^4^Department of Agricultural and Forestry Sciences, University of TusciaViterbo, Italy

**Keywords:** antioxidative defense system, *Cucurbitaceae*, molecular mechanism, root–shoot interaction, rootstock, *Solanaceae*, water stress

## Abstract

Drought is one of the most prevalent limiting factors causing considerable losses in crop productivity, inflicting economic as well as nutritional insecurity. One of the greatest challenges faced by the scientific community in the next few years is to minimize the yield losses caused by drought. Drought resistance is a complex quantitative trait controlled by many genes. Thus, introgression of drought resistance traits into high yielding genotypes has been a challenge to plant breeders. Vegetable grafting using rootstocks has emerged as a rapid tool in tailoring plants to better adapt to suboptimal growing conditions. This has induced changes in shoot physiology. Grafting applications have expanded mainly in Solanaceous crops and cucurbits, which are commonly grown in arid and semi-arid areas characterized by long drought periods. The current review gives an overview of the recent scientific literature on root-shoot interaction and rootstock-driven alteration of growth, yield, and fruit quality in grafted vegetable plants under drought stress. Further, we elucidate the drought resistance mechanisms of grafted vegetables at the morpho-physiological, biochemical, and molecular levels.

## Introduction

Globally, the demand for vegetables is increasing. Their cultivation has thus extended, especially in semi-arid and arid regions where recurrent drought and water shortages are quite common (Rouphael et al., 2012).

Drought depresses plant growth due to decreased cellular water potential and stomatal conductance, inhibition of photosynthesis, and enhanced reactive oxygen species (ROS) accumulation. This causes heavy and economically relevant yield losses (Altunlu and Gul, 2012; Liu et al., 2014). Thus, it is a considerable challenge to develop scientific strategies for minimizing yield losses under drought conditions (Cantero-Navarro et al., 2016), to increase water use efficiency (WUE) of crops. WUE is defined as the ratio of the CO_2_ assimilation rate and transpiration rate (instantaneous WUE), or the ratio of crop yield over the applied water (yield WUE). Grafting has emerged as a potential tool to quickly enhance the efficiency of modern vegetable cultivars for wider adaptability or resistance to different stresses when these are grafted onto resistant rootstocks for specific stresses (Colla et al., 2010b, 2011, 2012, 2013; Kumar et al., 2015a,b; Rouphael et al., 2016). In spite of widespread use of grafting in dealing with soil-borne diseases, recent research has demonstrated its potential in alleviating abiotic factors such as drought stress in different fruiting vegetables belonging to the *Solanaceae* and *Cucurbitaceae* families (Schwarz et al., 2010).

Understanding how grafting minimizes the effects of drought is a basic requirement for the continued success of this technique. The present article provides an updated review of scientific advances addressing grafting effects under drought conditions of fruiting vegetables and discusses the mechanisms that may mediate these effects.

## Agronomic Responses of Grafted Plants to Drought Stress

Drought resistance can be categorized as constitutive (also expressed under well-watered conditions) or drought-responsive (expressed only under pronounced water deficit) (Boopathi, 2012). Drought responsive traits affect yield only under severe water-stressed conditions while constitutive traits can influence yield at low and intermediate degrees of drought as well (Tuberosa, 2012). In a recent study, Liu et al. (2016) reported that luffa (*Luffa cylindrica* Roem. cv. Xiangfei No. 236) rootstock resulted in higher shoot growth (i.e., leaf area and dry weight) and instantaneous WUE, when grafted with its own scion or with cucumber (*Cucumis sativus* L. cv. Jinyan No. 4). Furthermore, it also showed delayed leaf wilting under water deficit conditions. Since no significant differences were observed on leaf area and plant dry weight between grafted and non-grafted plants under well-watered conditions, rootstock-induced drought resistance was probably the result of drought-adaptive response. Moreover, the use of reciprocal grafting between drought-tolerant luffa rootstock and sensitive cucumber indicated that plant resistance to drought was mainly dependent on luffa rootstock. Using reciprocal grafting between the drought-tolerant “Zarina” and sensitive “Josefina” tomato (*Solanum lycopersicum* L.) genotypes, Sánchez-Rodríguez et al. (2012a,b, 2013) reported that increased tolerance in grafted plants to moderate water deficit was a drought-adaptive response mainly related to tolerant rootstock, which provided better plant growth and yield. Moreover, Altunlu and Gul (2012) demonstrated that when the interspecific hybrid “Beaufort” (*S. lycopersicum* L. ×*S. habrochaites* S. Knapp and D. M. Spooner) was used as rootstock, it mitigated the growth depression of tomato plants induced by water stress, compared with tomato grafted onto weak root structure rootstock (“Resistar”) or self-grafted plants. Such findings indicate a drought-adaptive response of tomato grafted onto “Beaufort” rootstock. Comparing 144 tomato lines as rootstocks for grafting tomato “Boludo F_1_,” Albacete et al. (2015) found that 38% of lines exhibited higher shoot fresh weight compared to self-grafted plants under water deficit conditions. This finding confirmed that drought-induced growth inhibition in tomato can be alleviated by grafting. Similar to tomato, the marketable yield of greenhouse pepper (*Capsicum annuum* L. cv. Verset) was improved only under water-stressed conditions (drought-adaptive response) when grafted onto the rootstocks “Atlante,” “PI-15225,” and “ECU-973.” This was affected by their ability to maintain net photosynthetic rate under deficit irrigation (Penella et al., 2014a).

Rootstock-induced drought resistance can be the result of a constitutive response, also expressed under well-watered conditions. Ibrahim et al. (2014) and Al-Harbi et al. (2016) reported an increase of yield and yield WUE in tomato cv. Faridah grafted onto the interspecific tomato hybrid “Unifort” (*S. lycopersicum* L. ×*S. pimpinellifolium* L.), grown under both full and deficit irrigation regimes. A constitutive response of grafted plants (such as watermelon) to drought was also reported by Rouphael et al. (2008). They found higher marketable fruit yield (+60%) and yield WUE (+10%) in pumpkin rootstock (*Cucurbita maxima* Duch. ×*C. moschata* Duch.; cv. PS1313) grafted mini-watermelon [*Citrullus lanatus* (Thunb.) Matsum. and Nakai; cv. Ingrid] than non-grafted control, under full and deficit irrigation regimes. López-Marín et al. (2017) studied the morpho-physiological responses of greenhouse sweet pepper cv. Herminio non-grafted or grafted onto three hybrid rootstocks, “Atlante,” “Creonte,” and “Terrano,” under two irrigation regimes (100% or 50% of ETc). Pepper yield was higher in the three grafting combinations than in non-grafted plants, regardless of the irrigation regime used (constitutive response). Among the different rootstocks, “Creonte” was the most effective in improving yield and yield WUE of grafted plants. This experiment resulted in 25% higher marketable yield than non-grafted plants and ∼10% higher yield than the other two rootstocks. “Creonte” significantly increased the harvest index at both irrigation regimes in comparison with non-grafted plants without affecting shoot biomass. These findings indicated that “Creonte”-mediated increase in the partition of biomass to reproductive organs of grafted plants was the major trait associated with the improved performance of these plants under both irrigation regimes. The research findings of Penella et al. (2014a) and López-Marín et al. (2017), demonstrated that “Atlante” rootstock behaved differently depending on the cultivar used as the scion: a drought-adaptive response in the Atlante/Verset grafting combination and a constitutive response in the Atlante/Herminio grafting combination. The authors concluded that each graft combination has to be tested for drought tolerance, as both rootstocks as well as scion genotypes affect growth and yield response.

Petran (2013) reported that use of turkey berry rootstock (*S. torvum* Swartz) under water-stressed conditions induced dwarfness in tomato scions (“Celebrity” and “3212”) and delayed wilting, especially with “3212.” Similarly, grafting tomato cv. “BHN602” onto the tomato rootstock “Jjak Kkung” reduced the plant growth and leaf area under well-watered conditions in comparison with non-grafted or grafted plants BHN602/Cheong Gang (Nilsen et al., 2014). However, under drought conditions, the decrease in plant biomass and leaf area in BHN602/Jjak Kkung combination was less pronounced in comparison with non-grafted or grafted plants BHN602/Cheong Gang. The above findings indicate that the increase in drought resistance in tomato scions grafted onto turkey berry rootstock or tomato rootstock “Jjak Kkung” represents a drought-avoidance response. This characteristics has limited agronomic value because growth reduction (especially leaf area reduction), constitutively induced by rootstock, is often associated with lower yield potential.

The above research findings demonstrate that vigor (greater production of above-ground biomass in a short time) and high biomass partitioning in fruits (harvest index) are the major crop constitutive traits associated with high yield potential of watermelon (Ingrid/PS1313; **Supplementary Figure S1**) and pepper (Herminio/Creonte) grafted plants in both well-watered and drought stress conditions. As these traits are also expressed under well-watered conditions, it is possible to screen grafting combinations for vigor, harvest index, and yield in trials, where plants are grown only under a regular irrigation regime. Drought-adaptive response has been also reported in trials on cucumber, pepper, and tomato, and is often associated with a mitigation of yield decline only under severe water-stressed conditions. However, as areas under vegetable production are more likely to witness mild water shortage rather than severe water stress, it seems more promising to increase the phenotyping traits that constitutively enhance yield *per se* rather than plant survival under extreme drought.

Fruit quality of vegetable crops is also significantly affected by grafting combinations and water availability. Sánchez-Rodríguez et al. (2012a,b, 2013) reported that grafting sensitive “Josefina” tomato genotype onto drought-tolerant “Zarina” rootstock improved fruit quality, particularly with regard to sugars and organic acids, the ratio of sugar to acid, minerals (Ca, K, and Mg), lycopene, β-carotene, total flavonoids and rutin, under water-stressed conditions. However, other researchers (Turhan et al., 2011; Al-Harbi et al., 2016) have reported a decrease in fruit quality traits (vitamin C, titratable acidity, and total soluble solids) of grafted tomato plants. The disparities in the results regarding the effect of grafting on sweetness, acidity, and functional compounds may reflect the effects of different grafting combinations and environmental conditions on photosynthesis, source:sink ratio, water relationships, and fruit ripening time (Rouphael et al., 2010; Kyriacou et al., 2017).

For watermelon, Rouphael et al. (2008) reported an improvement of some fruit quality parameters (titratable acidity, K, and Mg) in pumpkin rootstock (*Cucurbita maxima* Duch. ×*C. moschata* Duch.; cv. PS1313) grafted mini-watermelon (*Citrullus lanatus* (Thunb.) Matsum. and Nakai; cv. Ingrid) than non-grafted control under full and deficit irrigation regimes. Moreover, antioxidant compounds (vitamin C and lycopene) in mini-watermelon were also higher in Ingrid/PS1313 grafting combination than in non-grafted control, irrespective of the irrigation regime used (Proietti et al., 2008). When averaged over irrigation regime, “Creonte” rootstock showed improved physical quality parameters (firmness) but reduced fruit nutritional value (vitamin C, antioxidant capacity, and total phenolic compounds) of pepper cultivar “Herminio,” compared to non-grafted plants. The above findings indicate that fruit quality attributes need to be considered during the selection process of drought resistance grafting combinations. This is true especially for watermelon and melon, where quality attributes such as total soluble solids determine sensory quality, and thereby, consumer acceptance (i.e., marketability).

A summary of the main agronomic effects of grafted plants in comparison to non-grafted or self-grafted plants in vegetable crops grown under full and deficit irrigation regimes is reported in **Table 1**.

**Table 1:** Agronomic response of the best performing grafting combinations in fruiting vegetable crops grown under full and deficit irrigation regimes.

Scion cultivar	Rootstock cultivar	Treatment factors^1^		Growing conditions	Agronomic response			Reference
		Grafting combination^2^	Irrigation regime		Growth and yield	Fruit quality	Water use efficiency	
Cucumber (*Cucumis sativus* L.) cv. Jinyan No. 4 (C)	Luffa (*Luffa cylindrica* (L) Roem.) cv. Xiangfei No. 236 (L)	Four grating combinations (C/C, C/L, L/C, and L/L)	Two irrigation regimes (well-watered to 50% substrate water content and drought induced by withholding irrigation)	Short term experiment (9 days) in pots filled with a 3:1 peat:vermiculite mixture under controlled environment (growth chamber)	Higher shoot dry weight and leaf area in C/L than in C/C under drought conditions		Higher instantaneous water use efficiency in C/L than in C/C under drought conditions	Liu et al., 2016
Pepper (*Capsicum annum* L.) cv. Verset (V)	Pepper (*C. annum* L.) cv. Atlante (A), C40 (C), Serrano (S), Tresor (T), NuMex Conquistador (N), Chili pepper (*C. chinense* Jacq.) accessions PI-152225 (P), and ECU-973 (E), *C. baccatum* var. *pendulum* accession BOL-58 (B)	Nine grafting combinations (ungrafted V, V/A, V/C, V/S, V/T, V/N, V/P, V/E, and V/B)	Two irrigation regimes (100%, and 50% of ETc)	Long term experiment (160 days) in a loam soil under greenhouse conditions	Higher fruit yield in V/A, V/P, and V/E than ungrafted control under water stress			Penella et al., 2014a
Pepper (*C. annum* L.) cv. Herminio (H)	Pepper (*C. annum* L.) cv. Atlante (A), Creonte (C), and Terrano (T)	Four grafting combinations (ungrafted H, H/A, H/C, and H/T)	Two irrigation regimes (100%, and 50% of ETc)	Long term experiment (224 days) in a clay loam soil under greenhouse conditions	Higher fruit yield in H/C, H/A, and H/T than ungrafted control across all irrigation regimes	Lower the antioxidant capacity in H/C and H/A, vitamin C in H/C, and total phenolic content in H/A, H/C, and H/T than ungrafted control across all irrigation regimes	Higher yield water use efficiency in H/A, H/C, and H/T than ungrafted control across all irrigation regimes	López-Marín et al., 2017
Tomato (*Solanum lycopersium* L.) cv. Faridah (F)	Interspecific tomato hybrid (*S. lycopersicum* L. x *S. pimpinellifolium* L.) cv. Unifort (U)	Two grafting combinations (ungrafted F, and F/U)	Four irrigation regimes (100, 80, 60, and 40% ETc)	Long term experiment (210 days) in a sandy soil under greenhouse conditions	Higher leaf area and fruit yield in F/U than ungrafted control across all irrigation regimes		Higher yield water use efficiency in F/U than ungrafted control across all irrigation regimes	Ibrahim et al., 2014
Tomato (*S. lycopersicum* L.) cv. Josefina (J)	Tomato (*S. lycopersicum* L.) cv. Zarina (Z)	Six grafting combinations (ungrafted J, ungrafted Z, J/Z, Z/J, Z/Z and J/J)	Two irrigation regimes (100%, and 50% field capacity)	Long term experiment (65 days) in pots filled with a 1:1 perlite: vermiculite mixture under controlled environment (growth chamber)	Higher fruit number and yield in J/Z than in the other grafting combinations under water stress	Higher total phenols, flavonoids, anthocyanins, lycopene, β-carotene, antioxidant activity, sugars and organic acids, sweetness index and sugars: acids ratio, Ca, K and Mg in J/Z than in the other grafting combinations under water stress		Sánchez-Rodríguez et al., 2012a,b
Tomato recurrent parent line (LA4024) (R)	Tomato introgression line LA3957 (*S. habrochaites* S. Knapp and D. M. Spooner line LA1777 introgressed into the genetic background of *S. lycopersicum* L. cv. E-6203 (LA4024)) (L)	Two grafting combinations (R/R, and R/L)	Two irrigation regimes (well-watered and drought induced by withholding irrigation)	Short term experiment (34 days) in pots under greenhouse conditions	Higher total plant dry matter and leaf area in R/L than other grafting combinations across all irrigation regimes			Poudyala et al., 2015
Tomato (*S. lycopersicum* L.) cv. BHN 602 (B)	Tomato (unknown tomato species) cv. Jjak Kkung (J) and cv. Cheong (C)	Three grafting combinations (ungrafted B, B/J, and B/C)	Two irrigation regimes (well-watered and drought induced by withholding irrigation)	Short term experiment (32 days) in pots filled with a commercial peat based substrate under greenhouse conditions	Reduction of leaf dry weight and leaf area with the increase of water stress in all grafting combinations except for B/J where no significant changes in leaf dry weight and leaf area were recorded			Nilsen et al., 2014
Watermelon (*Citrullus lanatus* (Thunb.) Matsum. and Nakai) cv. Ingrid (I)	Interspecific Cucurbita hybrid (*Cucurbita maxima* Duch. × *C. moschata* Duch.) cv. PS1313 (P)	Two grafting combinations (ungrafted I, and I/P)	Three irrigation regimes (100%, 75%, and 50% ETc)	Long term experiment (73 days) in a sandy loam soil under open field conditions	Higher above ground dry biomass and fruit yield in I/P than ungrafted control across all irrigation regimes	Higher lycopene, vitamin C, titratable acidity, and content of K and Mg in I/P than ungrafted control across all irrigation regimes	Higher yield water use efficiency in I/P than ungrafted control across all irrigation regimes	Proietti et al., 2008; Rouphael et al., 2008

*^1^Factorial designs with two-treatment factors (grafting combination and irrigation regime) were used in the cited experiments. ^2^First and second letter indicate the scion and rootstock, respectivetly.*

## Mechanisms of Drought Resistance in Grafted Plants

### Root System Characteristics

A deep root system has shown beneficial effects on plant production and survival by acquiring water stored in deeper soil layers, thus leading to more drought tolerance (Koevoets et al., 2016). Plants often reallocate assimilates from shoot growth to root growth under water-stressed conditions, thereby enhancing root extension into deeper soil layers (Xu et al., 2015). A strong and an extensive deep root system of pumpkin rootstock was a major contributor of drought resistance in grafted “Charleston Gray” watermelon (Poor, 2015). Similarly, López-Marín et al. (2017) found higher root growth in the drought-tolerant grafted sweet pepper plant Atlante/Herminio than in non-grafted pepper plant “Herminio” under an irrigation-deficient regime, which was associated with a higher leaf water content. In tomato, the use of drought-tolerant cv. Zarina as rootstocks for cv. Josefina increased root dry biomass and root metabolic efficiency, in comparison to self-grafted plants when water stress was imposed by reducing the irrigation to 50% of field capacity (Sánchez-Rodríguez et al., 2014). Recently, an introgression line (IL, “LA3957”) used as rootstock for grafting tomato resulted in a higher root dry matter and root to shoot ratio, besides showing better resistance to drought stress than self-grafted plants (Poudyala et al., 2015). Hence, we emphasize the importance of selecting grafting combinations that are able to develop a deep and vigorous root system, and of increasing the root to shoot ratio. Moreover, root anatomical characteristics (e.g., vessel size and density) and rooting hydraulic conductance may also play a pivotal role in increasing drought resistance of grafted plants. However, the use of roots as selection criteria of drought-resistance grafting combinations has a major limitation due to the difficulty of phenotyping root traits under field conditions.

### Physiological and Biochemical Mechanisms

#### Nutrient Uptake and Assimilation

It is well established that water stress decreases the plant ionome by restricting uptake and translocation of mineral nutrients owing to restricted transpiration rate and reduced active transport and membrane permeability (Sánchez-Rodríguez et al., 2013, 2014). Many rootstocks are capable of increasing the uptake and translocation of nutrients (Savvas et al., 2010). The increased fruit yield and yield WUE in grafted mini-watermelon plants under full and deficit irrigation regimes was partly due to the rootstock-mediated increased nutrient status of plants (i.e., N, K, and Mg; Rouphael et al., 2008). Accumulation of macro and micronutrients (N, P, K, Fe, and Cu) in susceptible tomato scion “Josefina” increased when grafted onto drought-tolerant “Zarina” rootstocks (Sánchez-Rodríguez et al., 2014). Working with similar plants, Sánchez-Rodríguez et al. (2013) concluded that drought-tolerant rootstock could improve absorption, upward transfer, and accumulation of NO_3_^-^ in tomato scion, thus stimulating nitrate reductase (NR) activity and NO_3_^-^ assimilation. Consequently, improved growth of grafted plants under moderate water stress was noted. Penella et al. (2014b) also observed that the NR activity in leaves of pepper plants increased when grafted onto tolerant rootstocks. The increase of nutrient uptake in drought resistance grafting combinations can be related to the considerable soil exploration resulting from the deep and vigorous root system, and the enhanced root exudation of organic acids into the soil, which contribute to the release of nutrients (e.g., P and Fe) more efficiently in the rhizosphere (Colla et al., 2010a). However, it may also be possible that rootstocks enhance nutrient transporters in the plasma membrane, thus increasing the capacity of grafted plants to take up nutrients.

#### Photosynthetic Efficiency and Water Relations

Increased WUE of grafted plants relative to non-grafted or self-grafted plants is often related to increases in net CO_2_ assimilation rate, or reduced transpiration rate, or both (Khah et al., 2011). In luffa rootstock-grafted cucumber, increased instantaneous WUE over self-grafted cucumber was due to a higher CO_2_ assimilation rate and lower transpiration rate in comparison to self-grafted plants under drought stress (Liu et al., 2016). Apart from stomatal movement, rootstock-induced changes in stomatal development also affects water conservation in grafted cucumber, as reduced transpiration relates to lower stomatal density in grafted plants (Liu et al., 2016). Compared to self-rooted mini-watermelon, the higher yield and yield WUE in grafted plants was due to their higher net CO_2_ assimilation rate, along with their efficiency in acquiring more water (high crop evapotranspiration) and nutrients from soil (Rouphael et al., 2008).

Drought resistance of grafted tomato plants onto rootstock “Beaufort” was due to improved osmoregulation, partially induced by higher proline content, and relative water content in tomato scion under water stress (Altunlu and Gul, 2012). Though net CO_2_ assimilation rate decreased in polyethylene glycol (PEG)-induced water stress, rootstock-grafted pepper seedlings maintained the protective capacity of the photosynthetic machinery mediated by osmotic adjustment (based on higher proline content; Penella et al., 2014b). Potassium plays a crucial role in osmoregulation and confers drought tolerance (Kafkafi and Xu, 1999). Sánchez-Rodríguez et al. (2014) observed an increase in the K-concentration under water-stressed conditions only in drought-tolerant rootstock-grafted plants (Zarina/Josefina) and non-grafted “Zarina,” which might have contributed to better osmotic adjustment under stress conditions.

Other than osmotic adjustment, grafted plants can maintain high leaf water potential under water-stressed conditions through drought avoidance mechanisms as reported by Nilsen et al. (2014) in tomato. In a former study, increased drought resistance in BHN602/Jjak Kkung grafting combination was observed to be the result of a water conservation strategy, based on the combination of growth reduction (reducing leaf area) and the ability to maintain net photosynthesis at lower water potentials. Similarly, an introgression line (IL, “LA3957”), used as rootstock for grafting tomato, resulted in a decrease of stomatal conductance in response to drought stress, so as to maintain leaf turgor (Poudyala et al., 2015). Moreover, the same graft combination showed higher stomatal conductance a few hours after the drought stress cycle break, allowing a rapid recovery of net assimilation. Furthermore, tomato plants (cv. Known-You 301) maintained higher leaf water potential and net photosynthesis when grafted onto *Solanum mammosum*, rather than self-grafted plants because of the higher hydraulic conductivity from roots to leaves (Weng, 2000). Overall drought resistance in some grafting combinations is achieved through a variety of adaptive traits involving the minimization of water loss (i.e., better control of stomatal movement and reduction of stomatal density) and optimization of water uptake (i.e., greater osmotic adjustment). Therefore, drought resistance-grafting combinations are able to carefully balance the ratio between uptake and loss of water in order to mitigate the detrimental effects of drought on net CO_2_ assimilation rate.

#### Antioxidative Defense System

Grafting with drought-tolerant rootstock can improve scion performance by regulating the antioxidant system under drought stress (Liu et al., 2014). The higher activity of antioxidant enzymes [superoxide dismutase (SOD) and catalase (CAT)] and lower oxidative stressors (hydrogen peroxide (H_2_O_2_) and malondialdehyde (MDA) content) from drought-tolerant tomato genotype “Zarina” (either grafted or self-rooted), indicate its greater ability to maintain cellular homeostasis, conferring plant drought resistance. Apart from SOD and CAT, increased levels of polyamines also displayed improved drought resistance mechanisms of grafted plants under water-stressed conditions (Sánchez-Rodríguez et al., 2016). Such mechanisms included acting as direct ROS scavenger, or scavenging free radicals through binding to antioxidant enzyme molecules (Groppa and Benavides, 2008). Luffa rootstock-grafted cucumber experienced lower stress than its self-grafted counterpart (Liu et al., 2016), possibly by inducing higher antioxidative enzymatic activities [e.g., SOD, dehydroascorbate reductase (DHAR) and guaiacol peroxidase (GPOD)] and ROS scavenging. Further, they presumed that resistance of luffa rootstock in grafted cucumber was also due to a partial contribution of abscisic acid (ABA) in ROS scavenging activity, possibly through ABA signaling and regulation of antioxidant reaction in response to drought. Nevertheless, increased H_2_O_2_ accumulation (in stomata) could possibly play a signaling role in rapid stomatal closure (Liu et al., 2016).

#### Hormonal Signaling

Abscisic acid is probably the most researched and crucial phytohormone inflicting shoot water relation via root-to-shoot signaling under water stress. Upward ABA transfer from roots to shoots influences transpiration rate (Wilkinson and Davies, 2002). Rootstocks with enhanced capacity for ABA biosynthesis or sensitivity can increase WUE and productivity of grafted scion under water stress (Liu et al., 2016). Luffa rootstock enhances instantaneous WUE of cucumber scion under drought stress. This was associated with the improved ability of luffa roots to sense changes in root-zone moisture, leading to a modest level of upward ABA transfer (Liu et al., 2016). Cantero-Navarro et al. (2016) observed that rootstock-mediated ABA was positively correlated with scion photosynthetic parameters and instantaneous WUE. However, ethylene precursor 1-aminocyclopropane-1-carboxylic acid (ACC) was negatively correlated with leaf growth and water use, but did not limit fruit yield. Hence, yield WUE in grafted tomato plants showed a marked increase. Reciprocal grafting study with ABA-deficient mutants (*flacca*) demonstrated that increased leaf area of scion/rootstock (*flacca*/wild-type parent; WT) over *flacca* self-grafts was not related to increased leaf-water relations. It was rather associated with the normalization of shoot ethylene relations, and possibly, direct promotion of growth by root-synthesized ABA (Dodd et al., 2009). Moreover, grafting experiments in tomato have postulated that rootstock-mediated regulation of stomatal conductance by ABA signaling may have a significant effect on tomato production under drought conditions (Nilsen et al., 2014). The role of other hormones classes (e.g., auxins, cytokinin, brassinosteroids, jasmonic acid, and salicylic acid) in regulating WUE by influencing plant water relation (Acharya and Assmann, 2009) under drought stress has yet to be clarified in grafted fruiting vegetables.

### Molecular Mechanisms

Liu et al. (2016) reported quantitative analyses of expression levels of the ABA signaling-related gene in water-stressed grafted and self-grafted cucumber plants. They observed a higher up-regulation in transcripts of the ABA receptors genes (PYL1, PYL2, PYL5, and PYL8), ABA transporter gene (ABCG22), ABA signaling-related genes (SnRK2.4 and SnRK2.5), and ABA-dependent genes (RAB18 and RD29B). Higher down-regulation of a negative regulator gene of ABA signaling (PP2C1) was also observed in drought-tolerant cucumber, grafted onto luffa rootstock, than drought sensitive self-graft cucumber. The above findings were in agreement with the increased ABA accumulation and cellular sensitivity to ABA in grafted plants (Liu et al., 2016). Therefore, it can be inferred that increased sensitivity of stomatal movement to ABA and water stress was associated with increased ABA accumulation and induction of the transcripts of ABA signaling genes. There is a need to study the function and implication of some other genes involved in accumulation of osmolytes, detoxification of ROS, and so on (Albacete et al., 2014), to decipher drought resistance in grafted plants.

## Conclusion

**FIGURE 1:**
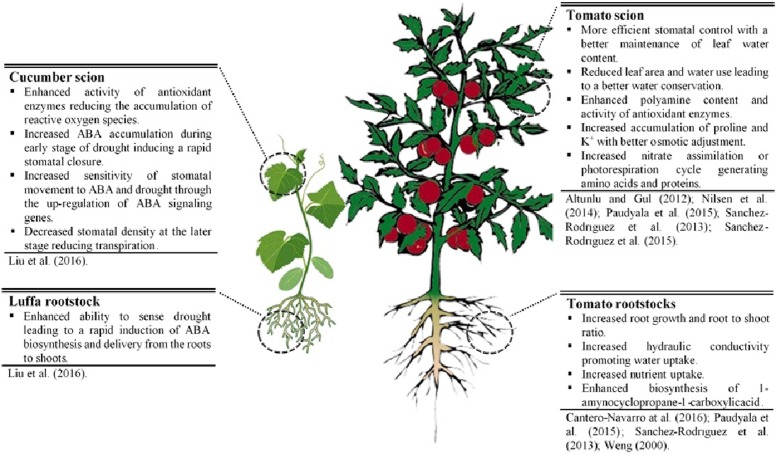
Physiological and molecular mechanisms underlying drought resistance in grafted cucumber and tomato plants.

The scientific literature reviewed here suggests that grafting can efficiently mitigate the adverse effects of drought on shoots of elite cultivar and increase WUE. Reported mechanisms for drought resistance of grafted plants (**Figure 1**) can bring about modifications in: (1) root traits (a deeper and more extensive root system, higher root hydraulic conductance, faster induction of hormone accumulation (like ABA)]; (2) scion-rootstock communication (water, nutrient, and hormonal flow); (3) scion morpho-physiological characteristics (increase in harvest index, decrease in leaf growth, reduction in stomata density, better osmoregulation, higher antioxidant activity, etc.). Selection of grafting combinations that constitutively increase morpho-physiological traits (e.g., scion vigor, harvest index, root growth, root hydraulic conductance, and nutrient uptake) associated with high yield potential, offers the most promising strategy to increase drought resistance in Solanaceous crops and Cucurbits under mild water-stressed conditions. Under extreme water-stressed conditions, a combination of constitutive and adaptive traits in grafting combinations could be ideally considered for improving drought resistance.

## Author Contributions

PK wrote the first draft of the manuscript; YR improved the manuscript and especially the agronomic part, MC improved the manuscript and especially the physiological part; GC supervised to the preparation of the manuscript and gave a substantial contribution in improving the different parts.

## Conflict of Interest Statement

The authors declare that the research was conducted in the absence of any commercial or financial relationships that could be construed as a potential conflict of interest.

## Supplementary Material

FIGURE S1 Early vigor of non-grafted (**A**; cv. Ingrid) and grafted plants (**B**; cv. Ingrid/PS1313) of mini-watermelon at the Experimental Farm of Tuscia University, Italy (30 days after transplanting). Pictures refer to the experimental trial reported by Rouphael et al. (2008).
