# Supplementary figures and images for: Vegetable Grafting as a Tool to Improve Drought Resistance and Water Use Efficiency
Source: Front Plant Sci. 2017 Jun 30;8:1130. doi: 10.3389/fpls.2017.01130 (PMC5492162; doi:10.3389/fpls.2017.01130)

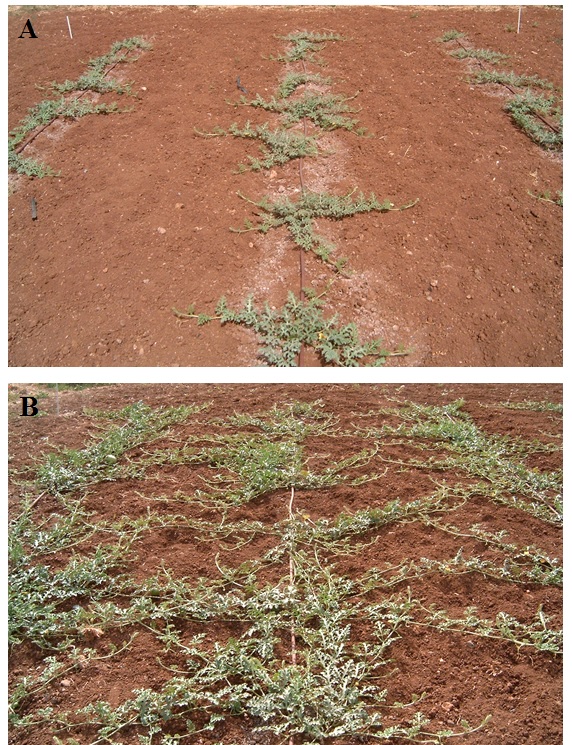

Supplement: FIGURE S1 — Early vigor of non-grafted (A; cv. Ingrid) and grafted plants (B; cv. Ingrid/PS1313) of mini-watermelon at the Experimental Farm of Tuscia University, Italy (30 days after transplanting). Pictures refer to the experimental trial reported by Rouphael et al. (2008). [file Image_1.JPEG]
